# Absorption Bands of Carbon Dioxide* From 5.3 to 4.6 Microns

**DOI:** 10.6028/jres.067A.020

**Published:** 1963-06-01

**Authors:** Arthur G. Maki, Earle K. Plyler, Robert J. Thibault

## Abstract

Measurements have been made of the frequencies of the infrared absorption lines of CO_2_ in the region from 1850 cm^−1^ to 2150 cm^−1^. Observations were made at various pressures and pathlengths up to a maximum of 72 meter-atmospheres. Vibration-rotation constants were obtained characterizing the transitions 11^1^*^c^*0–000, 11^1^*^d^*0–000, 03^1^*^c^*0–000, 03^1^*^d^*0–000, 200–01^1^*^c^*0, I2^2^*^c^*0–01^1^*^c^*0, 12^2^*^d^*0–01^1^*^d^*0 for C^12^O_2_. The 11^1^*^c^*0–000 band due to the C^13^O_2_ molecule was also measured.

## 1. Introduction

The infrared spectrum of CO_2_ has been exhaustively studied by Courtoy who has compiled a critical survey of the energy levels and vibrationrotation constants for the various isotopic species of that molecule [1, 2].^1^

The five micron region contains two weak absorption bands due to the transitions 03^1^0–000 and 11^1^0–000. These two bands form a Fermi resonant diad and are rather important for determining the best potential constants for CO_2_. Migeotte, Neven, Swenson, and Benedict have reported previous measurements on this region [3], but the resolution and wavelength accuracy available at that time was not quite as good as that now available in this laboratory. Because of slight disagreements between calculated and observed values, it was felt that those measurements should be repeated. In addition we have been able to resolve and analyze two *Q* branches for which the constants seem not to have been previously reported. The intensities of these bands are of great interest and of particular interest is the intensity anomaly which causes the *P* branch to be strong while the *R* branch is weak. Since this anomaly has already been discussed by Benedict in quantitative terms [4], it will not be further discussed here.

## 2. Experimental Procedure

The spectra were measured on the NBS high resolution infrared spectrometer described elsewhere [5]. Measurements were made using one of two gratings and a liquid nitrogen cooled PbSe detector. The highest resolution was obtained by using a 1,860 lines/in. grating double passed in third order. With this grating measurements were interpolated from a wavelength scale provided by measurements on a 7,500 lines/in. grating. Only lines that were not resolved with the finer grating were measured with the 1,860 lines/in. grating. Most of the measurements reported here were obtained directly from measurements with a 7,500 lines/in. grating used in the first order and double passed. Calibration with this grating was carried out by means of accurately measured rare gas spectra with a Fabry-Perot interferometer fringe system superimposed on the spectra for interpolation as described in reference 6.

The majority of the measurements were made with a pathlength of 24 m and pressures up to 1 atm. Pressures up to 3 atm were used in trying to observe lines due to the weaker transitions, but the increased pressure broadening of the lines usually resulted in a reduced sensitivity to the weaker lines. To achieve the highest resolution of the strong *Q* branches a 4 m path was used thus decreasing reflection losses in the absorption cell.

## 3. Analysis and Results

### 3.1. Analysis

Except for the *Q* branches the spectra were all analyzed using a least squares computer program to fit the observed frequencies to the power series

$$v_{\text{obs}}=v_{0}+(B^{\prime }+B^{\prime \prime })m+(B^{\prime }-B^{\prime \prime })m^{2}-2(D^{\prime }+D^{\prime \prime })m^{3}-(D^{\prime }-D^{\prime \prime })m^{4}$$

where the symbols have their usual meaning. The *Q* branch lines were fitted to the equation

$$v_{\text{obs}}=v_{0}+(B^{\prime }-B^{\prime \prime })J(J+1)-(D^{\prime }-D^{\prime \prime })J^{2}{\left(J+1\right)}^{2}.$$

The calculated and observed constants are tabulated in table 1. Where no observed constants are given, the data were insufficient to obtain those constants and the calculated values were used in the band analysis. Since it was felt that Courtoy’s values for *B*″ and *D*″ were more accurate than values obtainable from these measurements, Courtoy’s values, as given in reference 2, were used to determine the other constants for all the bands reported here.

The calculated values of *B*′ are those given by Courtoy [2] with the incorporation of a correction term due to the Coriolis interaction discovered by Courtoy and explained by Andrade e Silva and Amat [7]. The correction to the *B*′ terms was made in the manner described by Courtoy and Triaille [8]. This correction was quite small for these energy levels, the largest correction being about ‒1.4 × 10^−5^ cm^−1^ for *B*_12_^2^_0_.

The equations given by Nielsen, Amat, and Goldsmith [9, 10] were used to determine the approximate *l*-type resonance and Fermi resonance corrections necessary for the centrifugal distortion terms (*D_v_*). No attempt was made to include the effect of an additional *J(J+*1) dependence in the Fermi resonance matrix element which has recently been predicted by Maes [11]. Since the calculated values of Δ*D* are probably as good as the measured values, the calculated values of Δ*D* were used in determining the final values of Δ*B*. In those cases where a value of Δ*D* was experimentally determinable, both observed and calculated Δ*D*’s gave nearly the same Δ*B*’s.

In table 1 the standard deviations are given for the various constants. These deviations were determined solely by the scatter of the data and do *not reflect possible absolute errors*.

Table 2 and 3 list the observed and calculated frequencies (wavenumbers) for the absorption lines in the various bands. Where comparison is possible, the observed wavenumbers given in table 2 and 3 generally agree with those listed in reference 3 to within a tenth of a wavenumber (cm^−1^). For the least squares computations line measurements to three decimal places were used. Because of the doubtful significance of the third decimal place, the wavenumbers given in tables 2 and 3 have been rounded off to two decimal places.

### 3.2. C^13^O_2_ Band at 2037 cm^−1^

The band due to the 11^1^*^c^*0–000 transition of C^13^O_2_ was measured at 2037.09 cm^−1^. This band is part of a Fermi doublet of which the other band lies at about 1896.54 cm^−1^. The other band was not detected in this work even at the highest pressures and pathlengths used. It is expected to be considerably weaker than the 11^1^0–000 band due in part to the diminished Fermi resonance in C^13^O_2_. Consequently, it will probably be necessary to use isotopicly enriched samples before this 03^1^0–000 transition will be measurable. The *Q* branch for the 11^1^0 band of C^13^O_2_ was not resolved in this work. This is understandable since the Δ*B* term, which governs the *Q* branch line spacing, is predicted to be rather small and relatively large pressures were necessary to observe this weak band. Furthermore, this *Q* branch is overlapped by the *P* branch of the much stronger C^12^O_2_ band.

Table 1 gives a comparison of the calculated and observed values of the various vibration-rotation constants necessary to describe this band. The discrepancies in *v*_0_ and *B*′ are believed to be greater than the experimental error and are consequently indicative of either a very small error in Courtoy’s constants or the neglect of some terms in the potential expansion. Such errors are not unexpected for C^13^O_2_ as Courtoy did not have a surfeit of data from which to derive his constants. Table 2 lists the lines measured and the values calculated using the constants given in table 1.

### 3.3. CO_2_ Bands Between 1900 and 2150 cm^−1^

The strongest CO_2_ absorption bands in the 5*μ* region are those due to the Fermi doublet 03^1^0–000 and 11^1^0–000. For these transitions a rather large number of lines have been measured in the *P* and *R* branches and the *Q*, branches were sufficiently well resolved to yield good values for Δ*B*.

Two more bands due to the transitions 200–01^1^0 and 12^2^0–01^1^0 have been measured in this region. The latter band is split into *c* and *d* components. When treated individually the two components yield values *B*_12_^2^_0_ which should be equal and in fact are found to be the same to within 5×10^−6^ cm^−1^. Such agreement indicates that the standard deviations quoted are realistic. The values reported in table 1 for the 12^2^0–01^1^0 bands were obtained by assuming that the *c* and *d* components of the upper vibrational level had the same vibrational energy (band center) and *B_v_* values.

Similarly the analysis of the *c* and *d* components of each of the two Fermi doublet bands $3v_{2}^{1}$ and $v_{1}+v_{2}^{1}$ was carried out simultaneously so as to yield the the same values for the band centers of the *c* and *d* components.

The important constants *v*_0_ and Δ*B* for the Fermi doublet are in slight disagreement with the values predicted by Courtoy’s constants. At least in the case of *v*_1_+*v*_2_ this discrepancy is greater than experimental error. The band centers for the other bands in this region are in even greater disagreement with the calculated values.

No attempt has been made to reevaluate the various vibration-rotation constants in order to obtain a better fit with the data presented here. Such a task would be a major undertaking and Courtoy has indicated [8] that such work is in progress elsewhere. The fact that both *c* and *d* components of the two resonating diads have been resolved does allow one to make a direct evaluation of some combinations of constants.

For a resonance diad the sum of the *l* doubling terms (*q_v_*) for the two resonating energy levels is constant and independent of the degree of resonance. From table 1 one finds *q*_11_^1^_0_+*q*_03_^1^_0_=188.8×10^−5^ cm^−1^. Correcting for the interaction between the *c* levels and *v*_3_ this becomes 187.9×10^−5^ cm^−1^.

Since

$$q_{v}=\frac{1}{2}(v_{2}+1)\;[q_{0}-q_{1}v_{1}-q_{2}v_{2}-q_{3}v_{3}]$$

and

$$q_{01}{{\;}^{1}}_{0}=61\times 10^{-5}=q_{0}-q_{2}$$

then

$$q_{11}{{\;}^{1}}_{0}+q_{03}{{\;}^{1}}_{0}=183\times 10^{-5}-4q_{2}-q_{1}.$$

This gives 4*q*_2_+*q*_1_=−4.9×10^−5^ cm^−1^. Similarly the sum of Δ*B*_11_^1^_0–000_ and Δ_03_^1^_0–000_ yields −*α*_1_−4*α*_2_*+ γ*_11_ + 10*γ*_22_+*γ*_12_+2*γ_ll_* = 162.9 × 10^−5^ cm^−1^ when the Coriolis resonance correction is made. This compares very favorably with the value of 164.5×10^−5^ cm^−1^ calculated using the constants given on page 128 of reference 2. It is difficult to tell whether the difference of 1.6×10^−5^ cm^−1^ is significant.

The rather large deviations from the predicted positions of some of the band centers which has been found in this work and that of Plyler, Tidwell, and Benedict [12] may be due, at least in part, to the neglect of the constants *x*_1_*_ll_*, *x*_2_*_ll_*, and *x*_3_*_ll_* in the vibrational energy expansion. These constants are necessary for N_2_O [13] and have recently been found to be important for the vibrational analysis of HCN [14] and DCN.

## Figures and Tables

**Table 1:** Band constants for CO_2_ absorption bands between 1900 and 2150 cm^−1^ All values are given in wavenumbers (cm^−1^). The error limits given are standard deviations and do not reflect possible consistent errors which will affect the absolute values. Calculated values were obtained from constants given in reference 2

Assignment	C^13^O_2_ 11^1^*^c^*0–000	200–01^1^*^c^*0	12^2^*^c^*0–01^1^*^c^*0	12^2^*^d^*0–01^1^*^d^*0	03^1^*^c^*0–000	03^1^*^d^*0–000	11^1^*^c^*0–000	11^1^*^d^*0–000
								
*v*_0_ obs	2037. 093±0.006	2129. 769±0.005	2093. 356±0. 004		1932. 477±0. 004		2076. 890±0. 003	
*v*_0_ calc	2037.12	2129. 63	2093.29		1932.46		2076.85	
*B*″ calc	0.39025	0.39062	0. 39062	0. 39123	0.39021	0.39021	0. 39021	0. 39021
*B*′ obs	.39004	.39058_7_	.39151_2_	.39151_2_	.390723	.391677	.390377	.391311
*B*′ calc	.39011_1_	.39060_7_	.39153_6_	.39153_6_	.39070_2_	.39165_5_	.390454	.39134
*D*″ obs×10^8^	………	………	………	………	13.3±1.3	………	13. 6±1.1	………
*D*″ calc×10^8^	13.7	13.5	13.5	13.5	13.5	13.5	13.5	13.5
*D*′ obs×10^8^	12.3	………	………	………	14. 6	………	12.8	………
*D*′ calc×10^8^	12.8	9.8	10.5	12.4	14.7	15.2	12.4	11.9
Δ*B* obs	−0.00021±0.00002	−0.000033±0.000005	0. 000892±0. 000009	0. 000282±0. 000009	0.000513±0. 000002	0. 001467±0. 000004	0. 000167±0. 000002	0.001101 ±0.000003
Δ*D* obs×10^8^	1.4±0.7	………………	………………	………………	1.1±0.2	………………	−0. 7±0.1	………………

**Table 2:** Wavenumbers (cm^−1^) of weak absorption bands due to *C*^13^O_2_ and *C*^12^O_2_ in 5 micron wavelength region

*J*	C^13^O_2_ 11^1^*^c^*0–000		C^12^O_2_ 12^2c^0–01^1c^0				C^12^O_2_ 12^2^*^d^*0–01^1^*^d^*0				C^12^O_2_ 200–01^1^*^c^*0			
	*P*		*P*		*R*		*P*		*R*		*P*		*R*	
	Obs	Calc	Obs	Calc	Obs	Calc	Obs	Calc	Obs	Calc	Obs	Calc	Obs	Calc
1	………	………	………	………	2094.93	.92	………	………	………	………	………	………	………	………
2	………	………	………	………	………	………	………	………	2095.71	.71	………	………	………	………
3	………	………	………	………	96.51	.50	………	………	………	………	2127.42	.42	2132.91	.89
4	………	………	………	………	………	………	………	………	97.31	.28	………	………	………	………
5	………	………	………	………	98.07	.08	………	………	………	………	25.86	.86	34.49	.45
6	………	………	………	………	………	………	………	………	2098.85	.85	………	………	………	………
7	………	………	2087.91	.93	2099.66	.67	………	………	………	………	24.28	.30	………	………
8	………	………	………	………	………	………	………	………	2100.42	.42	………	………	………	………
9	………	………	86.37	.39	2101. 25	.27	………	………	………	………	22.71	.74	37. 56	.58
10	2029. 29	.27	………	………	………	………	………	………	02.01	.00	………	………	………	………
11	………	………	84.86	.86	………	………	………	………	………	………	21.17	.17	………	………
12	27. 69	.70	………	………	………	………	………	………	03. 59	.58	………	………	………	………
13	………	………	83.34	.34	………	………	………	………	………	………	19. 63	.61	2140.72	.70
14	26.13	.13	………	………	………	………	………	………	05.16	.16	………	………	………	………
15	………	………	81.85	.83	………	………	………	………	………	………	18.04	.05	………	………
16	24. 54	.56	………	………	………	………	………	………	2106.77	.74	………	………	………	………
17	………	………	80.31	.32	………	………	………	………	………	………	16.47	.48	………	………
18	23.00	.98	………	………	………	………	………	………	………	………	………	………	………	………
19	………	………	………	………	………	………	………	………	………	………	………	………	………	………
20	21.40	.41	………	………	………	………	………	………	………	………	………	………	………	………
21	………	………	………	………	………	………	………	………	………	………	13. 36	.36	………	………
22	19.83	.83	………	………	………	………	2076. 28	.28	………	………	………	………	………	………
23	………	………	75. 84	.85	………	………	………	………	………	………	11.79	.80	………	………
24	………	………	………	………	………	………	74.74	.74	………	………	………	………	………	………
25	………	………	………	………	………	………	………	………	………	………	10. 24	.24	………	………
26	………	………	………	………	………	………	73.21	.21	………	………	………	………	………	………
27	………	………	72. 90	.91	………	………	………	………	………	………	08. 68	.68	………	………
28	15.09	.10	………	………	………	………	71.68	.68	………	………	………	………	………	………
29	………	………	71.46	.46	………	………	………	………	………	………	07.16	. 12	………	………
30	13.51	.52	………	………	………	………	2070.17	.15	………	………	………	………	………	………

**Table 3:** Strongest *CO*_2_ absorption bands between 4.6 and 5.3 microns Line measurements are given in units of wavenumbers (cm^−1^)

*J*	03^1^*^c^*0–000				03^1^*^d^*0–000		11^1^*^c^*0–000				11^1^*^d^*0–000	
	*P*		*R*		*Q*		*P*		*R*		*Q*	
	Obs	Calc	Obs	Calc	Obs	Calc	Obs	Calc	Obs	Cale	Obs	Calc
												
0	………	………	………	………	………	………	………	………	………	………	………	………
1	………	………	………	………	………	………	………	………	………	………	………	………
2	………	………	1934.83	.82	………	………	2075. 30	.33	………	………	………	………
3	………	………	………	………	………	………	………	………	………	………	………	………
4	1929. 36	.36	36.39	.39	………	………	73. 75	. 77	2050. 81	.80	………	………
5	………	………	………	………	………	………	………	………	………	………	………	………
6	27. 82	.81	37. 98	.97	………	………	72.19	.21	82.37	.36	………	………
7	………	………	………	………	………	………	………	………	………	………	………	………
8	26.26	.26	39. 55	.55	………	………	70. 64	.66	83. 94	.93	………	………
9	………	………	………	………	………	………	………	………	………	………	………	………
10	24. 72	.72	………	………	………	………	69. 08	. 10	85.51	.50	………	………
11	………	………	………	………	………	………	………	………	………	………	………	………
12	………	………	………	………	………	………	67. 54	.55	87. 07	.07	………	………
13	………	………	………	………	………	………	………	………	………	………	………	………
14	21.65	.65	………	………	………	………	………	………	88. 62	.64	………	………
15	………	………	………	………	………	………	………	………	………	………	………	………
16	20.12	. 11	………	………	………	………	64. 43	.45	………	………	………	………
17	………	………	………	………	………	………	………	………	………	………	………	………
18	………	………	………	………	1932. 97	.98	………	………	91.73	.78	………	………
19	………	………	………	………	………	………	………	………	………	………	………	………
20	17.07	.07	………	………	………	………	61.35	. 35	………	………	………	………
21	………	………	………	………	………	………	………	………	………	………	………	………
22	15.56	.55	50. 73	.70	………	………	59. 80	.81	94. 93	.93	………	………
23	………	………	………	………	………	………	………	………	………	………	………	………
24	14.04	.03	52. 29	.31	33.32	.35	58. 26	.26	97. 50	.51	2077.56	. 56
25	………	………	………	………	………	………	………	………	………	………	………	………
26	12. 53	.52	53.91	.92	33.49	. 50	56. 71	.72	98. 07	.08	77. 68	.67
27	………	………	………	………	………	………	………	………	………	………	………	………
28	11.03	.02	55. 52	.53	33.65	. 66	55.17	.18	2099. 66	.66	77. 82	.79
29	………	………	………	………	………	………	………	………	………	………	………	………
30	09.53	.52	57.13	.15	33.83	.83	53.64	.64	2101. 25	.24	77. 94	.93
31	………	………	………	………	………	………	………	………	………	………	………	………
32			58. 75	. 77	34. 00	.01	52.11	.11	02. 83	.83	78. 08	.07
33	………	………	………	………	………	………	………	………	………	………	………	………
34	06. 54	.52	60. 37	.40	34.19	.20	50. 58	.58	04. 42	.41	78. 24	.22
35	………	………	………	………	………	………	………	………	………	………	………	………
36	05.05	.03	62.01	.02	34.40	.40	49. 05	.05	06. 00	.99	78.41	.39
37	………	………	………	………	………	………	………	………	………	………	………	………
38	03. 57	.55	63.64	.65	34.61	.61	47. 52	.52	07. 58	.58	………………	………………
39	………	………	………	………	………	………	………	………	………	………	………	………
40	02.08	.07	65. 28	.29	34.83	.84	46.01	.99	09.18	.17	78. 76	.74
41	………	………	………	………	………	………	………	………	………	………	………	………
42	1900. 61	.59	………	………	35.07	.07	44.47	.47	10. 77	. 76	78. 94	.93
43	………	………	………	………	………	………	………	………	………	………	………	………
44	1899.13	. 11	68. 54	.56	35.31	.31	42. 96	.95	12. 38	.35	………	………
45	………	………	………	………	………	………	………	………	………	………	………	………
46	97.66	.64	70.19	.20	35. 57	.57	………………	………………	13. 94	.95	………………	………………
47	………	………	………	………	………	………	………	………	………	………	………	………
48	96.19	. 17	71.82	.84	………………	………………	39. 94	.92	15. 57	.54	79. 54	.57
49	………	………	………	………	………	………	………	………	………	………	………	………
50	94. 73	.71	73. 46	.49	1936.13	. 11	38.41	.41	17. 16	. 14	79. 78	.80
51	………	………	………	………	………	………	………	………	………	………	………	………
52	93. 27	.25	75.12	. 13	………	………	36. 91	.90	18. 75	.74	2080. 05	.05
53	………	………	………	………	………	………	………	………	………	………	………	………
54	91.82	.79	76. 74	.78	………………	………………	35. 40	.40	20. 38	.34	………………	………………
55	………	………	………	………	………	………	………	………	………	………	………	………
56	90.32	.34	1978. 41	.43	………	………	33.91	.90	21.96	.95	………	………
57	………	………	………	………	………	………	………	………	………	………	………	………
58	88. 93	.89	………	………	………	………	32.40	.40	………	………	………	………
59	………	………	………	………	………	………	………	………	………	………	………	………
60	87.45	.44	………	………	………	………	30. 90	.91	25.16	. 16	………	………
61	………	………	………	………	………	………	………	………	………	………	………	………
62	1885. 99	.99	………	………	………	………	29.41	.42	2126. 76	. 77	………	………
63	………	………	………	………	………	………	………	………	………	………	………	………
64	………	………	………	………	………	………	27.91	.93	………	………	………	………
65	………	………	………	………	………	………	………	………	………	………	………	………
66	………	………	………	………	………	………	………	………	………	………	………	………
67	………	………	………	………	………	………	………	………	………	………	………	………
68	………	………	………	………	………	………	24.95	.98	………	………	………	………
69	………	………	………	………	………	………	………	………	………	………	………	………
70	………	………	………	………	………	………	………	………	………	………	………	………
71	………	………	………	………	………	………	………	………	………	………	………	………
72	………	………	………	………	………	………	22.04	.04	………	………	………	………
73	………	………	………	………	………	………	………………	………………	………	………	………	………
74	………	………	………	………	………	………	2020. 54	.58	………	………	………	………

## Footnotes

1 Figures in brackets indicate the literature references at the end of this paper.
